# My maize and blue brick road to physical organic chemistry in materials

**DOI:** 10.3762/bjoc.12.24

**Published:** 2016-02-08

**Authors:** Anne J McNeil

**Affiliations:** 1 Department of Chemistry and Macromolecular Science and Engineering Program, University of Michigan, 930 North University Avenue, Ann Arbor, Michigan 48109-1055, United States

**Keywords:** molecular gel, organic chemistry, organic materials, self-assembly, supramolecular chemistry

## Abstract

Similar to Dorothy’s journey along the yellow brick road in *The Wizard of Oz*, this perspective carves out the path I took from my early childhood fascinations with science through my independent career at the University of Michigan (maize and blue). The influential research projects and mentors are highlighted, including some fortuitous experimental results that drew me into the field of supramolecular chemistry, specifically, and organic materials, broadly. My research group’s efforts toward designing new sensors based on small molecule gelators are described. In particular, I highlight how our design strategy has evolved as we learn more about molecular gelators. This perspective concludes with some predictions about where molecular gels, as well as my personal and professional life, are headed.

## Review

One of my earliest memories involves pouring water down our driveway and watching the newly formed river break into small streams that later rejoined. Like most children, I was incredibly curious about the natural world around me. I set up terrariums and aquariums, used microscopes to examine leaves and insects, and kept spiders, snakes and turtles as pets. Although I sometimes dabbled with my brother’s chemistry set, I often found the simple experiments boring. At 15, I started working at the local library because I had an insatiable appetite for learning. Bringing home new reading material each night was worth the monotony of re-shelving books each day. It was here that I began exploring potential careers in astronomy, chemistry, ecology, and geology. At the time, there was no clear favorite and my uninspiring high school classes did little to tilt the balance. As I began considering college, I realized I was most interested in the chemical phenomena within each field. Learning that light emitted from stars comes from hydrogen fusing to form helium, or that a pond’s carbonate concentration affects buffering capacity and health, or that traces of iron in quartz lead to the purple color of amethyst, was all fascinating to me. It is cliché, but I recognized the centrality of chemistry in the natural sciences and, as a result, I wanted to learn more.

At the College of William and Mary (W&M) my appreciation for chemistry grew as I took classes from engaging professors, including Dr. Gary Rice and Dr. Trevor Hill. Dr. Rice wore shorts and t-shirts to class, cracked jokes, amazed us with fire-laden and/or explosive demos, and made balancing equations interesting. Dr. Hill had the opposite persona; the (untrue) rumor was that he invented Teflon while working at DuPont, was incredibly wealthy, and taught purely for the love of chemistry. The aura that surrounded him was palpable and he did not disappoint. Because of him I began to see organic chemistry in the world around me, and I was hooked. My father still reminisces about the time I spent our three-hour ride home describing the molecular basis behind the stretchiness of rubber bands, among other things. I could not stop talking and we both knew that I had found my passion.

**Figure 1:**
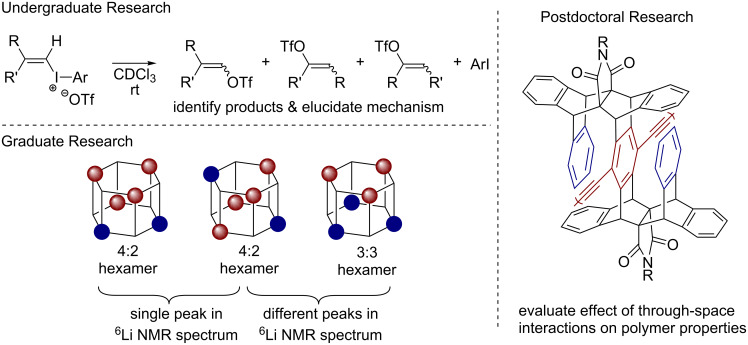
Summary of research experiences prior to independent career.

With encouragement from my faculty advisor, Dr. Debbie Bebout, and second-semester organic chemistry professor, Dr. Rob Hinkle, I began doing independent research. I was fortunate that Rob agreed to take me on for what turned out to be a three-year research experience. My initial goal was to identify six isomeric fragmentation products that were first observed by former student Dave Thomas (Figure 1). Once identified, I was tasked with elucidating the mechanism(s) that led to those products. What drew me into the lab was applying what I learned in class (e.g., substituent effects) to this unknown research question. I was driven by the desire to collect new data, and provide new information about the reactivity of these compounds [1–2]. Rob was a great mentor and role model; he had high expectations for himself and worked hard to achieve them. I was fortunate to have another great, albeit unofficial, mentor at W&M, Dr. Carey Bagdassarian. He was creative and passionate, and he encouraged, supported, and pushed me to be a better person and scientist. I left W&M feeling prepared for graduate work, and excited about the opportunity to gain even more breadth and depth in organic chemistry.

Entering graduate school at Cornell, I wanted to continue using physical organic chemistry principles to solve chemical mysteries. Although Cornell had a great selection of professors doing both fundamental and applied physical organic chemistry, I was most interested in working with Professor Dave Collum. He was known for unraveling complex mechanisms using a seemingly simple combination of kinetic, spectroscopic, and computational studies. I was fortunate, and will be forever grateful, that Dave accepted me into his group. We embarked on a project aimed at understanding why a lithium enolate alkylation stalled at 70% conversion during a key step in the preparative scale synthesis of a factor Xa inhibitor at Aventis. At the time, identifying solution structures of lithium enolates by NMR spectroscopy was challenging owing to the absence of Li–O coupling and the high symmetry of most common aggregates. Dave suggested we examine nonracemic mixtures to break symmetry (e.g., R_2_S_2_ versus R_3_S_1_ tetramers). At low temperatures, however, we saw just two major signals: one peak for the 100% R (and 100% S) aggregate and another peak for a heterochiral 50:50 R/S aggregate. We suspected a dimeric aggregate because our rate studies revealed a first-order dependence on enolate concentration. A major breakthrough occurred when Dave saw spectroscopic data wherein the “baseline junk” emerged as two additional resonances on warming; he excitedly declared that the “junk” was instead the complexity of higher aggregates. Sure enough, the “junk” showed a dependence on optical purity consistent with hexameric enolates (Figure 1). We recruited physics graduate student Gil Toombes to help us fit the data using an iterative parametric method and what emerged was a beautiful story [3–5] (and method) that is still being used to determine the aggregation state of lithium enolates [6–9] and alkoxides [10–13]. From Dave, I learned a tremendous amount about being a scientist. One of the most important take-home messages was that all data, whether intentionally collected or not, is part of the story. Dave taught me how to be an efficient experimentalist, how to write papers and grants, and to have a critical eye for everything that you read in the literature. Dave also showed me how to balance an academic career with having a family. He was a great mentor who knew exactly when to push, when to provide assistance, and when to disappear and let me figure it out on my own.

A fortuitous and unusual observation during my graduate work led me into the field of organic materials: I observed an enolate alkylation wherein the rate correlated with how fast it stirred. Because I was measuring rates by quenching independent (but supposedly identical) reactions at various times, I first suspected that the stir-rate effect was due to the initial mixing of reagents depending on each vial’s position in a grid on my stir plate. Once I measured the rate in a single round-bottom flask I realized that the stir-rate effect was real. I eliminated obvious culprits, including heterogeneity and high viscosity. Puzzled, I talked about this oddity to whomever would listen and tried every experiment suggested. One day I let a reaction sit unstirred for an hour while I attended a seminar; when I returned, the reaction mixture had formed a gel. In the end, we hypothesized that faster stirring disrupted more supramolecular aggregates, providing additional reactive sites for the alkylation, thereby accelerating the rate.

As I read more about gels and other organic materials, I began to see the field of physical organic chemistry more broadly. For my postdoctoral studies, I wanted to learn more about how (macro)molecular structure influences a material’s solution and solid-state properties. I was drawn to Professor Tim Swager’s research based on his creativity in (macro)molecular design and applied work with conjugated polymers. Tim pitched ideas for dozens of projects and let me decide where to focus. It took a few months and a few failed projects before I identified a clear research direction. In fact, one of those failed projects led to a new idea: to evaluate the effect of through-space (rather than through-bond) interactions on a conjugated polymer’s properties. During this project I synthesized some of the most beautiful molecules I have ever seen, with one and two cofacial arenes surrounding the central arene of the monomer (Figure 1) [14]. The synthetic chemistry was elegant and simple. In the end, we saw substantial through-space effects on the monomer’s properties, which were diminished in the polymer. Nevertheless, the cofacial arenes provided a physical, protective barrier from oxygen, and as a result, we observed reduced photobleaching, which is problematic for solid-state applications of conjugated polymers. Working with Tim I learned that new materials are interesting if they offer new properties, a lesson that still influences my research today. I learned how to manage my efforts, including when to stop working on unsuccessful projects. Tim’s biggest impact, however, was on my presentation style. Tim critiqued my 10 minute “best poster” Gordon Research Conference talk for over an hour. Every little pixel, color, and bond was discussed. It was an eye-opening experience that has had a long-lasting impact. I am forever grateful for his time and advice.

While on the academic interview circuit, I was fortunate to have Steven Wheeler (a postdoctoral researcher with Professor Ken Houk at the time) in the audience at one interview. He was developing computational methods to evaluate π-stacking interactions, and was intrigued by our surprising substituent effects on the Diels–Alder regioselectivity in our monomer synthesis. Together we designed a collaborative project to further evaluate these effects. These studies led us to conclude that the π system is relatively unimportant and that substituent effects can instead be explained by through-space interactions [15]. Steven, who is now an associate professor at Texas A&M University, is changing the way we understand π stacking, XH/π, and ion/π interactions in organic systems [16–20].

**Figure 2:**
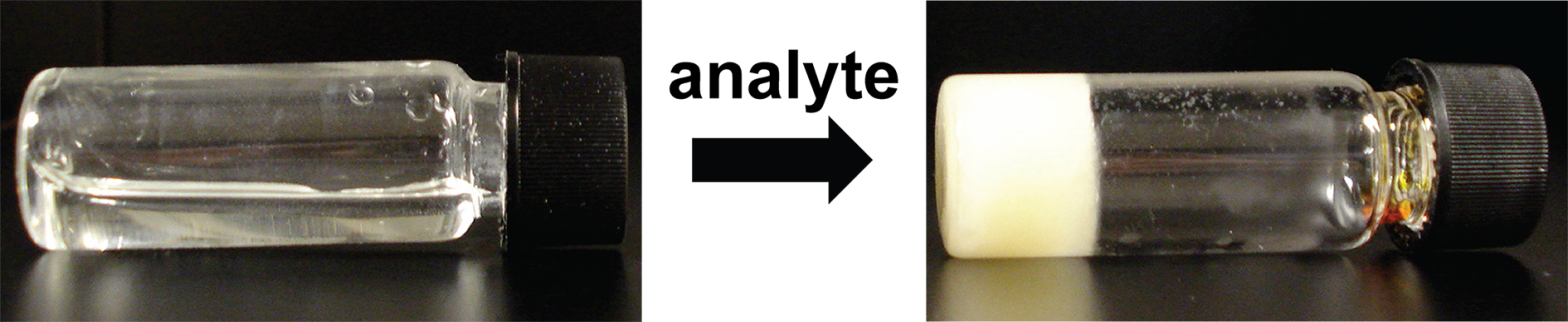
Sensing via analyte-triggered gelation.

During my postdoctoral studies, I remained fascinated with gels and was inspired by the creative work of many researchers in the field at the time [21–24]. When it came time to assemble a set of job proposals, it seemed natural for one focused on molecular gelation. Specifically, I proposed to develop sensors wherein a chemical stimulus (analyte) reacts with a “latent gelator” and induces gelation (Figure 2). Gel-based sensors were appealing because they provide an unambiguous visual change in the material’s physical properties with no interference from colored or opaque samples. Moreover, no instrumentation or training is necessary to interpret the results, thereby providing a portable and potentially inexpensive method for sensing. Considering how naïve my understanding of gelation was at the time, I am still surprised that my proposal idea worked almost exactly as described.

Molecular gels form through the self-assembly of small molecules into supramolecular structures, such as ribbons, fibers, and sheets. This self-aggregation is driven by noncovalent interactions, including hydrogen bonding, π stacking, van der Waals interactions, and halogen bonding. Physical interactions amongst these larger structures lead to gel formation. Because noncovalent interactions are involved, gel formation is reversible and can respond to environmental changes. Understanding which molecules will form gels and under what conditions remains a significant challenge. As a consequence, new gelators are often “discovered” by modifying known gelator scaffolds. Although successful, this approach is limited to existing scaffolds and specific solvents, which may not be suitable for every application.

**Figure 3:**
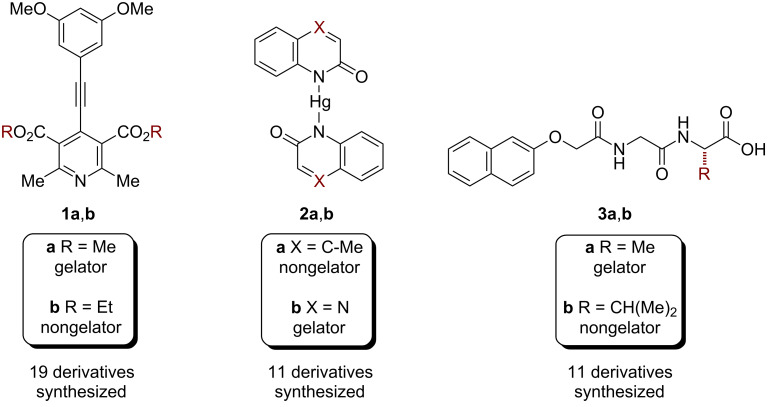
Examples of structurally similar gelators and nongelators examined in our studies.

Our work with molecular gels began with two research questions: (1) How can we accurately predict which molecules will form gels? (2) How can we develop sensors where an analyte triggers gel formation? One of my first graduate students, Jing Chen, evaluated the use of an oxidation reaction to convert a nonplanar molecule into a planar one [25]. We hypothesized that this conformational change, combined with an increase in conjugation length, would facilitate self-assembly and gelation via π stacking. This hypothesis was based on Hanabusa’s suggestion that unidirectional (1D) intermolecular interactions are necessary for gelation [26]. Excitingly, we found our first gelator (**1a**) after synthesizing just three molecules (Figure 3). Witnessing our first gel form remains one of my career highlights. Further characterization revealed that the π-stacking direction was coincident with the long axis of the fiber, providing support for the 1D interaction hypothesis. The original goal was to use nitric oxide (NO) as the oxidant, because a high concentration of NO in exhaled breath correlates with many diseases [27]. The NO-triggered oxidation and gelation worked, but there were a few limitations. Because NO was largely insoluble in the gelling solvent, we had to sequentially add NO and then additional solvent. When catalytic quantities of NO were used, the reaction rates were too low for sensing in real time. Increased NO concentrations led to gels within a minute, but these concentrations were outside the useful range for breath analysis. Nevertheless, these initial studies laid the foundation for our next effort, which was focused on developing a more sophisticated approach to gelator design.

We hypothesized that the Cambridge Structural Database (CSD), which contains over 700,000 organic and inorganic crystal structures, could be used to identify molecules that exhibit 1D interactions in the solid state. The rationale was that these molecules, or closely related derivatives, might be gelators. We searched the CSD for molecules containing a mercury atom (Hg^2+^) that was involved in an intermolecular cation–π interaction [28]. We identified molecule **2a**, which exhibited 1D π–cation–π interactions in the solid state (Figure 3). Graduate student Kelsey (King) Carter synthesized just three compounds before gelator **2b** was discovered. Single crystals of **2b** revealed a surprising 1D π-stacking interaction, rather than the expected cation–π interaction. Nevertheless, powder X-ray diffraction (PXRD) analysis revealed that the packing within the single crystal was not representative of the packing within the gel. As a consequence, the gel structure remains unknown. Instantaneous gelation is observed when adding Hg-contaminated water to a solution containing the ligand, albeit at high concentrations of Hg^2+^. We further demonstrated that gelation removes >98% of the Hg^2+^ from the contaminated water, leading to potential applications in environmental remediation [24].

In both studies we observed that seemingly minor changes in structure have a surprisingly strong effect on gelation ability. For example, converting a methyl ester (**1a**) to an ethyl ester (**1b**), which was not expected to affect the 1D intermolecular interactions, made the difference between a gelator and nongelator (Figure 3). Intrigued by the role of structure in gel formation, we asked ourselves, “What is special about the gelators compared to the structurally quite similar nongelators?” To address this question, we synthesized a large group of gelators and nongelators, with the long-term goal of elucidating their unique properties.

In our first effort, we synthesized 19 pyridine-based compounds, wherein 8 were gelators and 11 were nongelators [29]. One hypothesis from the literature was that gelators are “not too soluble or too insoluble” [30–31]. In contrast, we found gelators at both the low and high ends of the solubility spectrum (0.001 to 1 mg/mL), alongside the nongelators. We then probed the hypothesis by Hanabusa regarding the importance of 1D intermolecular interactions [22]. We were able to obtain single crystals of six gelators and five nongelators. Of the six gelator crystal structures, only three had PXRD patterns that matched the gel. Within this limited data set we found 1D intermolecular interactions being present and absent amongst both gelators and nongelators, providing no clear distinction based on molecular packing. Next, we performed a Hirshfeld surface area analysis to quantitatively evaluate the intermolecular interactions within the crystal structures [32]. This analysis provides information about the nature and extent of intermolecular interactions in the solid state. Surprisingly, three nongelators exhibited Hirshfeld surface areas similar to the gelators, suggesting the types of intermolecular interactions (e.g., van der Waals, H-bonding, π-stacking) were similar amongst the gelators and nongelators. Because this analysis involves counting interactions without weighting them according to their influence on the solid-state structure, we began investigating alternative measures of intermolecular interactions.

**Figure 4:**
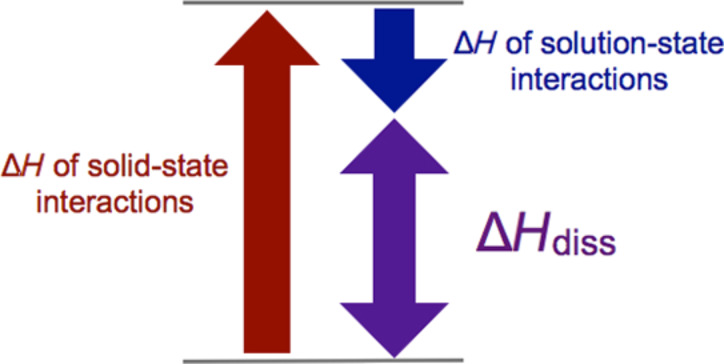
Relationship between dissolution enthalpies and intermolecular interactions. Gelators exhibit (on average) larger Δ*H*_diss_ than nongelators, suggesting stronger solid-state interactions and weaker solvent interactions.

We hypothesized that gelation might instead depend on the strength of intermolecular interactions in the solid state. To test this hypothesis, graduate student Jing Chen measured dissolution enthalpies (Δ*H*_diss_) of both the gelators and nongelators by determining their solubility at various temperatures [25]. The dissolution enthalpy reflects both the solid-state gelator/gelator interactions as well as the solution-state gelator/solvent interactions (Figure 4). Examining all 19 compounds revealed that gelators had higher dissolution enthalpies than the nongelators (on average), suggesting that the gelators had stronger solid-state gelator/gelator interactions and/or weaker gelator/solvent interactions. When comparing the same compounds in a different solvent system, similar trends were observed. To determine whether these results were general, postdoctoral researcher Maria Muro-Small synthesized and measured dissolution enthalpies for 11 dipeptide-based compounds (6 gelators, 5 nongelators) [33]. Peptides were chosen because they represent the largest and most widely investigated class of molecular gelators and their gelation ability is highly dependent on their sequence (e.g., **3a** versus **3b**, Figure 3) [34]. We again observed the trend that gelators exhibit higher dissolution enthalpies. During these studies, we discovered that several dipeptides underwent solid–solid transformations during the solubility measurements. Graduate student Kelsey (King) Carter later observed similar solid–solid transformations with our Hg complexes, precluding further analysis [35]. Ultimately, our take-home message was that strong intermolecular interactions and weak solvent interactions are important in gelation.

At this point, we wanted to develop a method that could predict solid-state dissolution enthalpies, rationalizing that such an approach could be useful for identifying new gelators. Graduate student Cheryl Moy ambitiously learned molecular mechanics simulations with mentorship from my colleague Professor Charles L. Brooks III [36]. Our goal was to model the solid-state interactions as well as the solvent interactions. We wanted to avoid starting the simulation with a crystal structure, knowing that this criterion would ultimately limit the structural diversity, so we modeled the solid-state as a liquid. Unfortunately, starting from a random, liquid orientation resulted in gelators and nongelators being enthalpicly indistinguishable. Using the crystal lattice as input provided better estimates of the dissolution enthalpies, but the gelators and nongelators within this limited data set remained indistinguishable.

**Figure 5:**
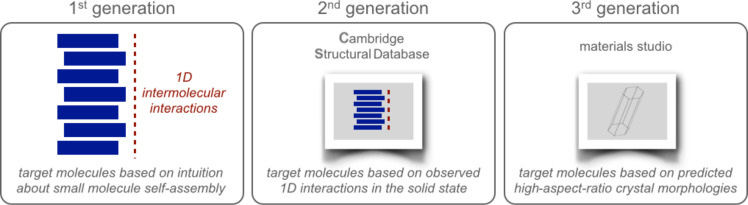
Evolution of our design strategy for identifying new gelators.

**Figure 6:**
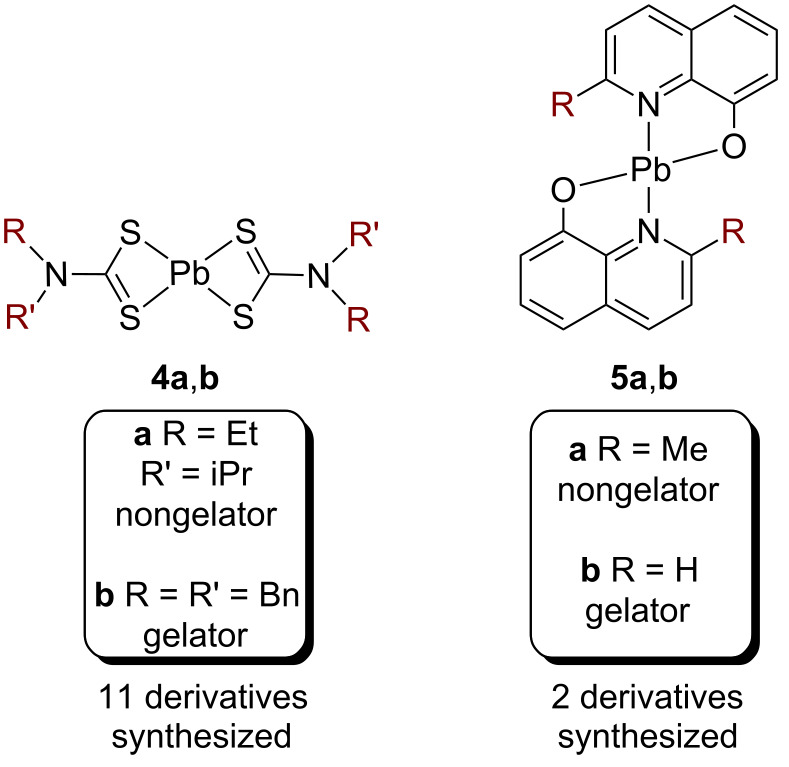
New gelator scaffolds identified by predicting crystal morphologies.

We then returned to our original CSD approach and modified it to incorporate our new understanding about the importance of strong intermolecular interactions (Figure 5) [37]. Specifically, we hypothesized that the driving forces for anisotropic growth in crystals and gel fibers might be similar. Because needle-shaped crystals form when the intermolecular interactions in one dimension are significantly stronger than the others, we predicted that molecules with needle-shaped morphologies (or their closely related derivatives) might be gelators. To test this hypothesis, we used crystal morphology prediction tools to identify needle-shaped crystals in the CSD. Graduate student Kelsey (King) Carter and undergraduate student Sarah Cox predicted the morphologies of 186 Pb(II)-containing crystals. Focusing on the top 5% and selecting stable and easily synthesized compounds (e.g., **4a** and **5a**), we discovered two new gelators (**4b** and **5b**, Figure 6). Postdoctoral researcher Gesine Veits synthesized nine additional derivatives and found both gelators and nongelators. These exciting results suggested that the driving forces for forming high-aspect-ratio crystals and gel fibers may be similar and guided by strong, directional intermolecular interactions. Overall, this approach for identifying new gelators should be generalizable, and therefore useful for developing new molecular gel-based applications.

**Figure 7:**
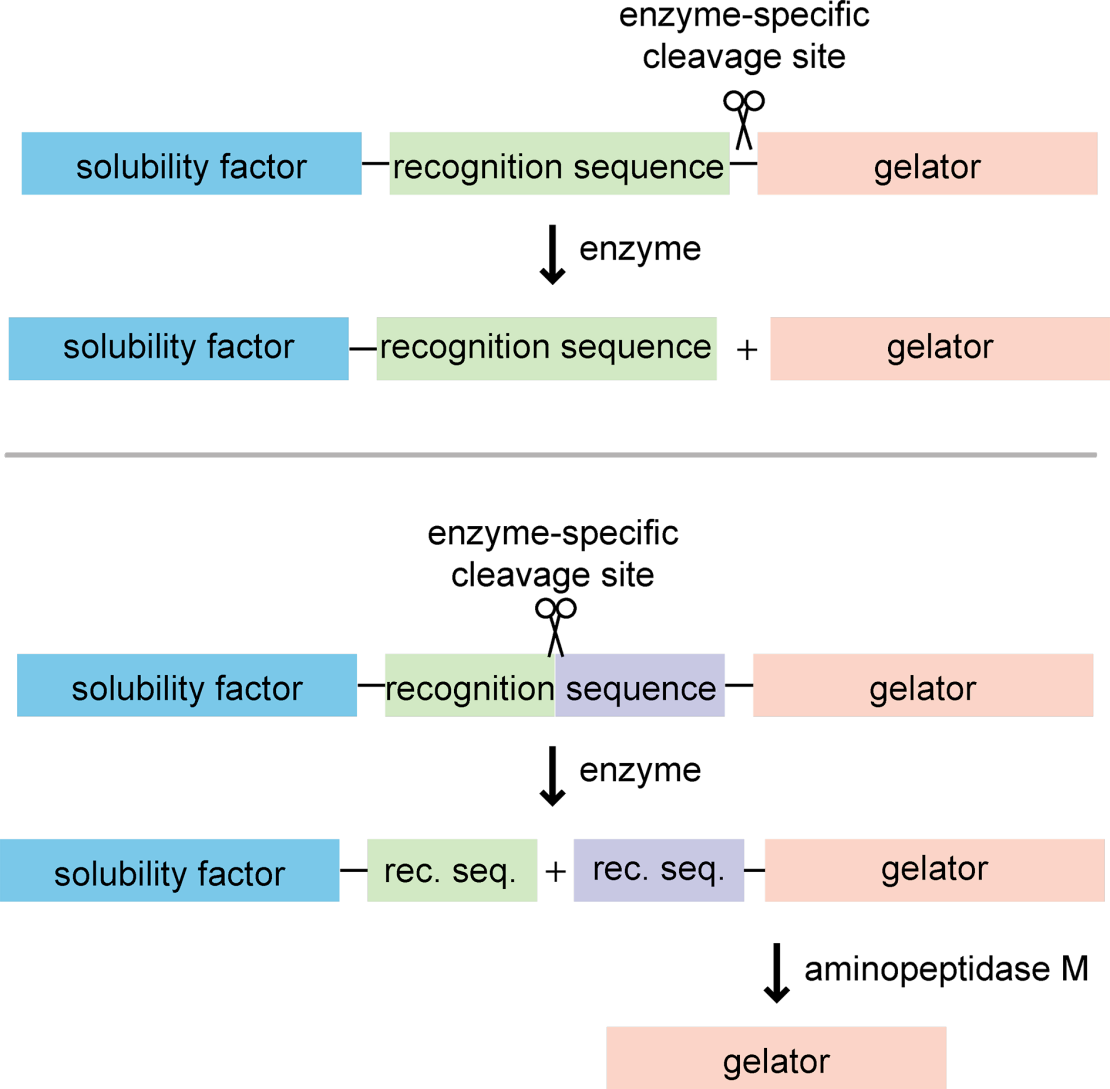
Two complementary approaches for sensing protease activity using gel formation.

Alongside these fundamental studies, we were interested in applying our new gelators in sensing platforms. Because analyte-mediated sensors rely on a chemical transformation to take place prior to gel formation, the reaction rate should be fast, or ideally instantaneous. While most of our sensors were designed with this criterion in mind, we were sometimes surprised to find slower and/or lower yielding reactions than reported. In these cases, we evaluated the stability of the reaction intermediates [38], measured reaction rates for both the desired and undesired products [39] and optimized the conditions to accelerate the desired transformations [34–35]. We learned from our early efforts that generating highly sensitive sensors required analytes that were efficient catalysts. With postdoctoral researcher Steven Bremmer and collaborator Professor Matt Soellner, we targeted gelation-based sensors using enzymes as the analytes [40–41]. Enzymes were attractive analytes because many diseases are correlated with their overactivity and/or overexpression. Specifically, we selected proteases, which play important roles in many biological processes, including blood clotting, apoptosis, and pathogenesis. Prior to our work in this area, there were several examples of enzyme-triggered gelation [42–44]; however, most of these systems were not responsive to physiological enzyme concentrations and not generalizable. We hypothesized that an enzyme-triggered cleavage that separates a recognition sequence from a gelator would represent a general and modular strategy for detecting proteases (Figure 7) [36]. The key advantage of this system is that simply swapping out the recognition sequence can lead to gelation-based sensors for other proteases. We applied this method to three different proteases and demonstrated that gelation could occur under physiological enzyme concentrations. We later generalized the approach even further to include proteases with internal cleavage sites (Figure 7) [37].

Additionally, we began investigating alternative ways to improve the sensitivity of gelation-based sensors for noncatalytic analytes. Graduate students Cheryl Moy and Danielle Zurcher evaluated “disassembling” polymers wherein an analyte-mediated end-group cleavage triggers depolymerization, releasing many gelators for each analyte [32,45]. We investigated three different polymeric scaffolds but have not yet successfully polymerized any monomer that forms a gel when released. We briefly investigated several methods of increasing sensitivity by lowering the critical gel concentration (cgc) using external (nongelator) modifications. For example, we decreased the cgc by lowering the solvent volume and/or reducing the vial diameter [35]. In a different example, we hypothesized that polymeric additives, commonly utilized to alter crystallization processes, might be useful for lowering the cgc in gel formation. Graduate student Yash Adhia, working with undergraduates Tracy Schloemer and Maria Perez, screened a series of commercially available polymers and discovered that adding poly(acrylic acid) (PAA) led to an incredible 90% reduction in the cgc of **1a** [46]. We determined that PAA was adsorbing onto the gel fibers during growth, which decreased the growth rate, leading to thinner fibers that were either longer or greater in number. These morphological changes reduced the cgc likely through additional physical entanglements from the longer and/or more prevalent fibers. Combined, these results suggested that additives may be a simple method to modify gel formation without having to alter the molecular structure.

**Scheme 1:**
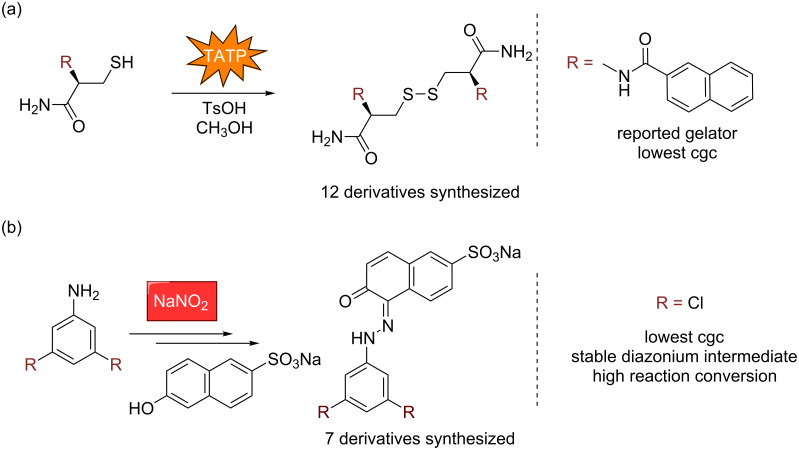
Sensors based on modifying known gelator scaffolds.

We have also explored the more conventional approach of modifying a known gelator to identify a new gelator with a lower cgc [34–35]. Graduate student Jing Chen, working with undergraduate Weiwei Wu, developed a sensor to detect milligram quantities of the explosive triacetone triperoxide (TATP) [35]. We synthesized 12 derivatives and found just three additional gelators; however, none of them exhibited lower cgcs than the original, reported gelator (Scheme 1a). Graduate student Danielle Zurcher, working with undergraduate Julian Díaz Romero, developed a sensor for nitrite contamination in water sources [34]. In this case, five additional compounds were synthesized, all of which were gelators, with some exhibiting lower cgcs than the known gelators (Scheme 1b). Given the mixed results of this approach, we believe that our CSD-morpholgy prediction method represents a better strategy for identifying new gelators for specific applications moving forward.

**Figure 8:**
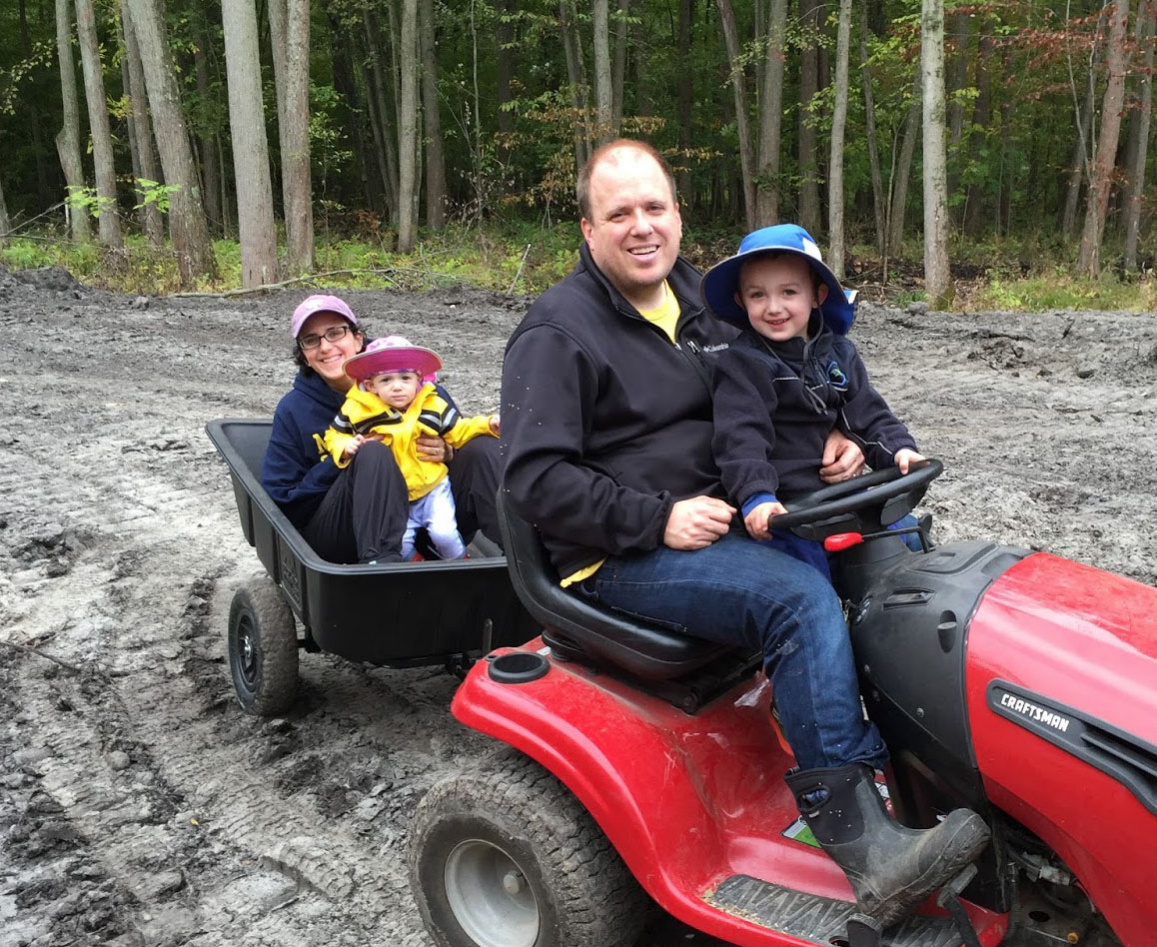
Enjoying the outdoors with my family, especially when it involves mud! Photo credit: Donald A. McNeil.

Although I started my independent career just eight years ago, so much has changed in my life, both personally and professionally. I met my wonderful husband (Professor Matt Soellner), got married and now have two awesome children (Evie and Emily). I am happiest when we are outside, exploring our world together (Figure 8). Professionally, I have taken on new leadership roles at the University of Michigan and elsewhere. My lifetime love of learning has led me down a path to better understand how other people learn. This path involved starting a funded research program in chemistry education. I am grateful that I can continue exploring new research areas as a professor. I have also realized that, like the library of my youth, there is no better place for me than the University of Michigan. I am surrounded by an amazing group of colleagues within my department and on campus. I am especially grateful for my mentors here at the University of Michigan: Professors Brian Coppola, Carol Fierke, Adam Matzger, Tim McKay, Melanie Sanford, and John Wolfe, who each have played a significant role in my professional development. I am looking forward to seeing what additional changes will occur in my family and career in the future.

## Conclusion

Overall, we have developed a strategy for discovering new gelators that focuses on molecules exhibiting strong, 1D intermolecular interactions in the solid state. This approach is simple to execute and expected to be generalizable. We anticipate that this new design strategy will transform gelator discovery from its current random screening to a more rational approach. With this streamlined approach to identifying new gelators, both the number and utility of molecular gel-based applications should increase. In addition, we have performed both fundamental and applied studies toward gelation-based sensors for a diverse set of analytes. Many of the strategies described herein can be used to improve other gel-based applications. Moving forward, we plan to capitalize on our experiences by embarking on a new (to us) research direction: exploring gels as templates for synthesizing materials with high surface areas.

The future of molecular gels remains bright. Two areas of growing importance include: (i) applying advanced solid-state characterization methods to gain insight into the molecular packing within gel fibers, and (ii) efforts towards correlating gelation ability with solvent properties (e.g., Hansen solubility parameters). These studies can provide further insight into gelator/gelator interactions as well as solvent/gelator interactions. Analogous to our efforts, these studies need to move from a post-experiment rationalization to a predictive model to be useful for gelator design. It is here where computational methods can and should play a significant role.
